# Effect of Chromium Content on the Passive Film and Corrosion Behavior of Steel Reinforcement in a Simulated Concrete Pore Solution

**DOI:** 10.3390/ma19101939

**Published:** 2026-05-08

**Authors:** Haipeng Lu, Yuwan Tian, Danmei Wu

**Affiliations:** Guangdong Provincial Ocean Equipment and Manufacturing Engineering Technology Research Center, School of Mechanical Engineering, Guangdong Ocean University, Zhanjiang 524088, China; 13318236459@139.com (H.L.); tianyuwan90@163.com (Y.T.)

**Keywords:** Cr, steel reinforcement, concrete, corrosion, chloride

## Abstract

Chloride-induced corrosion of steel reinforcements is one of the main factors restricting the durability of reinforced concrete structures. Chromium (Cr) alloying is an effective strategy to enhance the corrosion resistance of steel. However, the appropriate Cr content for different environments remains undetermined. In this study, steels with three different Cr contents of 0, 5, and 10 wt.% were prepared. Electrochemical methods and physical characterization techniques were used to investigate the effects of Cr content on the passive film and corrosion behavior of steels in a simulated concrete pore solution under chloride attack. The results show that Cr alloying increases the critical chloride concentration for steel depassivation, passive film resistance, and charge transfer resistance. Specifically, the critical chloride concentrations of 0Cr, 5Cr, and 10Cr are 0.63, 0.81, and 1.56 mol/L, respectively. In a simulated pore solution with 0.6 mol/L chloride, the charge transfer resistances of 0Cr, 5Cr, and 10Cr are 4.1, 5.8, and 63.4 × 10^5^ Ω·cm^2^, respectively, corresponding to corrosion rates that are 1.39- and 15.31-times lower for 5Cr and 10Cr relative to 0Cr. Therefore, in concrete exposed to marine chloride attacks, the use of high Cr alloying is necessary. Although the cost increases and the weldability deteriorates, the improvement in corrosion resistance is far superior to that of medium Cr alloying. The excellent corrosion resistance of high-Cr steel stems from its passive film mainly composed of stable Cr_2_O_3_ with a lower oxygen vacancy defect density, while that of 5Cr is dominated by less stable Cr(OH)_3_, which weakens the corrosion resistance of the passive film.

## 1. Introduction

Reinforced concrete structures, with advantages such as excellent mechanical properties and low cost, are widely used in many fields including construction, bridges, and marine engineering. Their service life directly determines the safety and durability of engineering structures. However, during long-term service, concrete is vulnerable to environmental factors such as carbonation and chloride ingress. These factors can cause the depassivation of the steel reinforcement, thereby triggering electrochemical corrosion. The corrosion process can reduce the cross-sectional area of the steel reinforcement, cause cracking of the concrete cover, and deteriorate its mechanical properties. This corrosion issue of steel reinforcement not only significantly increases the engineering maintenance cost but also may poses serious safety hazards [1,2,3,4].

Chromium (Cr), as an efficient corrosion-resistant alloying element, is widely used in the modification and preparation of corrosion-resistant steel reinforcements. Its main mechanism is the ability to form a dense and stable passive film on the steel surface, effectively impeding the penetration of corrosive media and electron transfer, thus significantly enhancing the corrosion resistance of steels [5,6,7]. Liu et al. systematically investigated the corrosion resistance of low-Cr steel (from 0% to 5 wt.%) in cement extraction solutions and a simulated concrete pore solution [8,9,10,11]. They found that Cr can effectively inhibit the oxidation of passive film from Fe^2+^ to Fe^3+^. Moreover, Cr enriches in the inner layer of the passive film, forming a dense Cr-Fe composite oxide layer, enhancing the stability of the passive film. Ai et al. [12,13,14] systematically studied the corrosion behavior of Cr10Mo1 steel in the simulated concrete pore solution and obtained conclusions similar to those of low-Cr steel. In conclusion, numerous studies have confirmed that with the increase in Cr content, the corrosion resistance of steel exhibits a gradually improving trend [15,16,17]. This phenomenon is mainly attributed to the fact that Cr can promote the formation of a protective passive film with reduced defect density, thereby enhancing the physical and chemical barrier effect against corrosive species and delaying the initiation of steel depassivation.

However, the Cr alloying of steel also gives rise to some adverse effects in engineering. Although the corrosion resistance of steel is consistently enhanced with the increase in Cr content, certain key engineering properties, such as weldability and cold working performance, will significantly deteriorate. Simultaneously, a high Cr content elevates the production cost. These adverse effects have spurred the development of low/micro alloyed steels. Nevertheless, existing research predominantly focuses on low-Cr steels and high-Cr steels separately, and only a few have studied the quantitative enhancement of corrosion resistance in both low- and high-Cr steels in the same environment, restricting their practical engineering applications.

The aim of this study is to explore the corrosion behavior and the micro-characteristics of the passive film of low-Cr steel and high-Cr steel in a concrete environment, aiming at the quantitative selection of Cr-alloyed steels in practical engineering. Specifically, this study measures the critical chloride concentrations of different Cr-alloyed steels in simulated concrete pore solution. Then, the effect of Cr content on the semiconductor properties and chemical composition of the passive film are studied through electrochemical techniques and X-ray photoelectron spectroscopy (XPS) techniques. Finally, the internal correlations among the Cr content, the characteristics of the passive film, and the corrosion resistance to chloride-induced depassivation are established.

## 2. Materials and Methods

### 2.1. Materials

The chemical compositions and labels of the steel reinforcement are shown in Table 1. The C content of carbon steel is 0.25 wt.% to ensure mechanical strength. For Cr-alloyed steels, due to the solid-solution strengthening effect of the Cr, the carbon content is slightly lower. Other chemical reagents, such as epoxy resin, sodium chloride, calcium hydroxide, etc., were purchased from Aladdin (Shanghai, China) and Macklin (Shanghai, China). Deionized water was used in the experiments.

### 2.2. Electrochemical Tests

All steels were cut into cubes of 10 × 10 × 5 mm^3^. Copper wires were welded to the steel cube and then encapsulated with epoxy resin. The working surface was polished with 400# sandpaper until the scratches were uniform. Subsequently, the samples were immersed in a simulated concrete pore solution, composed of saturated calcium hydroxide and sodium chloride. In each batch of experiments, the solutions were prepared collectively to ensure the consistency of environmental factors such as pH value and dissolved oxygen content. Before each test, the solution stood for 5 min to stabilize the oxygen level, and the pH value was measured by a pH meter. Electrochemical experiments were carried out using a three-electrode system with a Huachen CHI660E electrochemical (Chenhua Instrument Company, Shanghai, China) workstation. The steel sample was used as the working electrode, a platinum sheet as the counter electrode, and a saturated calomel electrode (SCE) as the reference electrode. All electrochemical tests were conducted at room temperature (25 ± 3 °C). The average value of three parallel samples was taken, and the error was calculated.

In order to measure the critical chloride concentrations of different Cr-alloyed steels, the samples were immersed in a saturated calcium hydroxide solution for pre-passivation treatment for 168 h. After that, 0.015 mol/L sodium chloride was added every 24 h to simulate the chloride-contaminated concrete environment. The open-circuit potential (OCP) [7] and electrochemical impedance spectroscopy (EIS) [18] were measured daily or every ten days. EIS was measured under OCP, with an amplitude of 10 mV and a frequency range of 10^−2^ to 10^5^ Hz. All EIS data were fitted using ZSimpWin software (v3.60).

In order to test the corrosion rates, the pre-passivated steels were immersed in a mixed solution of saturated calcium hydroxide and 0.6 or 4 mol/L sodium chloride; then, EIS was measured, and the charge transfer resistance was analyzed.

In order to test the semiconductor characteristics of the passive film, the pre-passivated steels were immersed in a saturated calcium hydroxide solution for Mott–Schottky tests. The test frequency was 1 kHz, the amplitude was 10 mV, and the potential range was from −1.5 to 0 V_SCE_.

### 2.3. XPS Tests

The chemical composition and chemical states of the passive films after being immersed in a saturated calcium hydroxide solution for 168 h were characterized using a Thermo escalab 250XI XPS instrument (Waltham, MA, USA). The characteristics of the passive films formed in a saturated calcium hydroxide and 0.6 mol/L NaCl solution were also characterized. The X-ray source was monochromatic Al Kα (hv = 1486.6 eV), with a power of 150 W, a beam spot of 650 μm, a voltage of 14.8 kV, and a current of 1.6 A. The binding energy was calibrated using C_1s_ = 284.8 eV, and the results were fitted with Avantage software (v6.92).

## 3. Results

### 3.1. Critical Chloride Concentration

**Figure 1:**
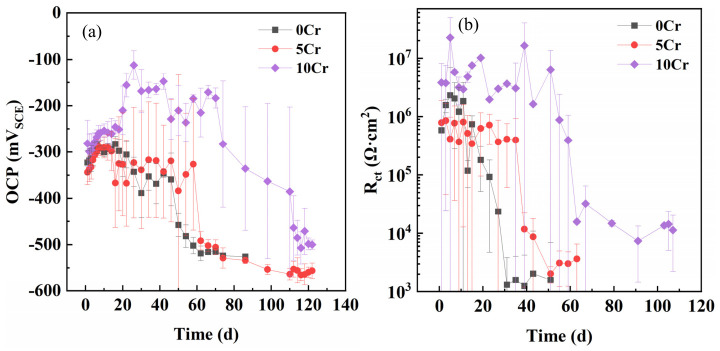
OCP evolution curves (**a**) and charge transfer resistance evolution curves (**b**) of the three different Cr-alloyed steels in a saturated calcium hydroxide solution with the gradual addition of chloride ions.

After three types of steels with different Cr contents were immersed in a saturated calcium hydroxide solution for 168 h, chloride ions were gradually added until the steels were depassivated. OCP and EIS experiments were carried out either daily or every ten days. As shown in Figure 1, when the chloride concentration was relatively low, the OCP values of all samples increased every day, and a high charge transfer resistance was maintained. At this time, the OCPs were above −300 mV_SCE_, and the charge transfer resistance was above 5 × 10^5^ Ω·cm^2^. When the chloride concentration reached a certain value, the OCP and charge transfer resistance dropped sharply (the slow decrease in the OCP in Figure 1 was due to taking the average value of three parallel samples). At this time, the OCP was below −400 mV_SCE_, and the charge transfer resistance dropped below 5 × 10^4^ Ω·cm^2^. According to the standard ASTM C876-22b [19], when the potential of uncoated steel bars in concrete is greater than −200 mV_CSE_, the probability that the steels do not corrode is greater than 90%, while when the potential of the steel bars is lower than −350 mV_CSE_, the probability that the steels corrode is greater than 90%. Since the potential difference between CSE and SCE is approximately 75 mV, the above detected potential values are reasonable.

The critical chloride concentrations of 0Cr, 5Cr, and 10Cr are 0.63 mol/L, 0.81 mol/L, and 1.56 mol/L, respectively. Compared with 0Cr, the critical chloride concentrations of 5Cr and 10Cr are increased by 1.286 and 2.476 times, respectively. Therefore, the improvement in chloride-induced corrosion resistance from 0Cr to 5Cr is not significant, while the increase from 5Cr to 10Cr is substantial. The charge transfer resistances of the three steels in the passive region are approximately 7.6 × 10^5^ Ω·cm^2^, 4.35 × 10^5^ Ω·cm^2^, and 4.293 × 10^6^ Ω·cm^2^, respectively. Evidently, the resistance of 5Cr in the passive state is similar to that of 0Cr, or even slightly lower, while the resistance value of 10Cr is 4.59 times higher than that of 0Cr. This indicates that the equilibrium dissolution rate of the 5Cr passive film is not much different from that of 0Cr, while the dissolution rate of the 10Cr passive film decreases significantly. In the active region, the charge transfer resistances of 0Cr, 5Cr, and 10Cr are approximately 1.9 × 10^3^ Ω·cm^2^, 3.2 × 10^3^ Ω·cm^2^, and 12.8 × 10^3^ Ω·cm^2^, respectively. That is, the corrosion rates of 5Cr and 10Cr are decreased by 1.69 and 6.64 times compared with 0Cr, respectively. This suggests that 10Cr can only undergo active corrosion at a higher chloride concentration, and even when active corrosion occurs, its corrosion rate is still nearly one order of magnitude lower than that of 0Cr.

### 3.2. Corrosion Rate

**Figure 2:**
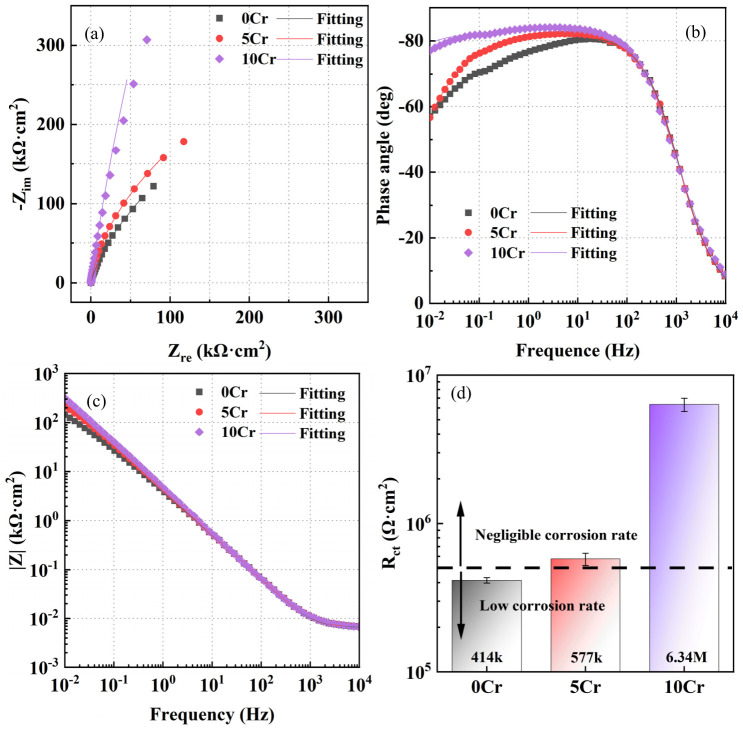
EIS spectra of three steels with different Cr contents after being immersed in a saturated calcium hydroxide solution for 168 h and then adding 0.6 mol/L sodium chloride: (**a**) Nyquist plot, (**b**) phase–frequency plot, (**c**) magnitude–frequency plot, and (**d**) charge transfer resistance, R_ct_.

After three types of steels with different Cr contents were immersed in a saturated calcium hydroxide solution for 168 h, 0.6 mol/L of sodium chloride was added, and following an additional 12 h immersion, the EIS spectra were measured (Figure 2). Evidently, all steels exhibit similar characteristics of electrode reactions. The Nyquist plots are characterized by a capacitive arc with an extremely large radius [20,21]. In the Bode plot, the broad peak of the phase angle implies that two time constants exist [22]. The order of the capacitive arc radius, phase angle peak value, and low-frequency impedance modulus follows the same trend: 10Cr > 5Cr > 0Cr, indicating that the corrosion resistance increases with the Cr content in the steel. The R_s_(Q_f_(R_f_(Q_dl_R_ct_)))-equivalent circuit was used to fit the EIS data, where R_s_ is the solution resistance, which reflects the resistance to ion transport in the corrosive medium; Q_f_ is the constant phase element of the passive film, used to describe the non-ideal capacitive characteristics of the film layer, and its derived parameter n_f_ can be used to evaluate the uniformity of the film layer; R_f_ is the passive film resistance, which is directly related to the film thickness, density, and defect density, characterizing the protective efficiency of the passive film; Q_dl_ is the constant phase element of the double layer, representing the non-ideal capacitive characteristics of the double-layer at the metal/passive film and medium interface, and n_dl_ reflects the interface stability; R_ct_ is the charge transfer resistance, corresponding to the resistance to electron transfer at the interface during the corrosion reaction, and is negatively correlated with the corrosion rate [23]. At the electrochemical corrosion interfaces, structural undulations, uneven roughness, and irregular defect distribution exist in the passive film. The double layer at the metal/solution interface is also non-uniform due to the influence of corrosion active sites and adsorbed species. These facts cause the capacitance characteristics to deviate from the ideal state, and therefore, the interface capacitance is characterized by a constant phase angle element (CPE), instead of an ideal capacitor C. The ideal capacitance values for the passive film (*C_f_*) and the double layer (*C_dl_*) were calculated using the following equations [24]:

(1) $$C_{f}\;=\;{Q_{f}}^{\frac{1}{n}}\;\times \;{R_{f}}^{\frac{1-n}{n}}$$

(2) $$C_{\mathrm{dl}}={Q_{\mathrm{dl}}}^{\frac{1}{n}}\;\times \;{\left(\frac{R_{s}R_{\mathrm{ct}}}{R_{s}+R_{\mathrm{ct}}}\right)}^{\frac{1-n}{n}}$$

The EIS fitting results from Figure 2 are shown in Table 2. It can be seen that the R_f_ of 5Cr increases significantly compared with that of 0Cr, rising from 9.7 × 10^3^ Ω·cm^2^ to 1.52 × 10^5^ Ω·cm^2^. When the Cr content is further increased to 10 wt.%, R_f_ slowly increases to 2.83 × 10^5^ Ω·cm^2^. Therefore, the higher the Cr content, the greater the impedance of the passive film, and the better the resistivity and protectiveness. Increasing the Cr content by only 5 wt.% can significantly increase the passive film resistance, while a further increase in Cr content reduces its rising rate. R_ct_ reflects the kinetics of the electrochemical corrosion process, that is, the dissolution rate of the steel substrate. The R_ct_ of 0Cr is the lowest (4.14 × 10^5^ Ω·cm^2^). The R_ct_ of 5Cr is 5.77 × 10^5^ Ω·cm^2^, which is 1.4 times higher than that of 0Cr. The R_ct_ of 10Cr is 6.34 × 10^6^ Ω·cm^2^, showing the most significant increase, 15.8 times higher than that of 0Cr. Thus, the higher the Cr content, the greater the charge transfer resistance, and the slower the substrate dissolution rate. The improvement efficiency of the corrosion rate of 10Cr compared with 5Cr is much higher than that of 5Cr compared with 0Cr.

**Figure 3:**
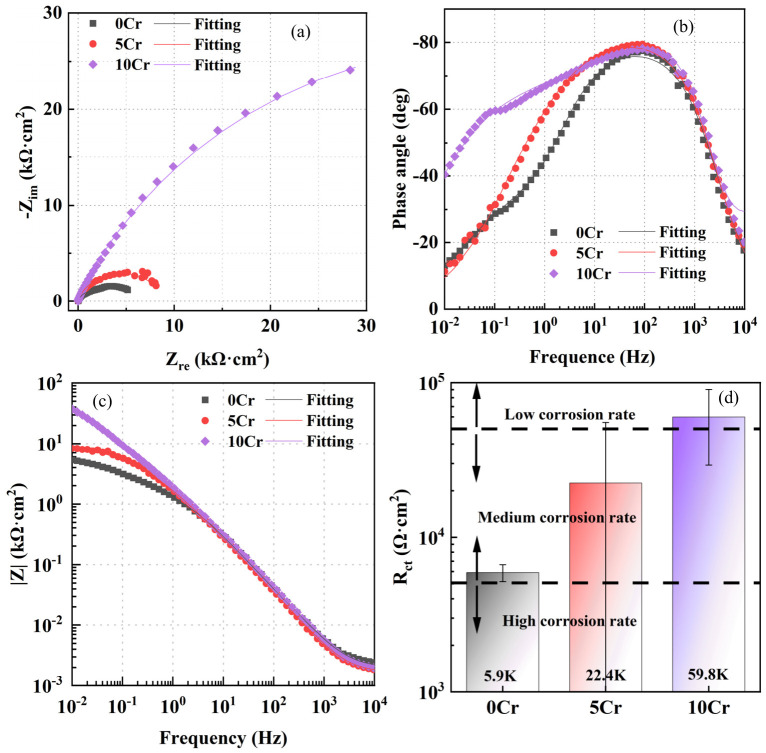
EIS spectra of three steels with different Cr contents after being immersed in a saturated calcium hydroxide solution for 168 h and then adding 4 mol/L sodium chloride: (**a**) Nyquist plot, (**b**) phase–frequency plot, (**c**) magnitude-frequency plot, and (**d**) charge transfer resistance, R_ct_.

After three types of steels with different Cr contents were immersed in a saturated calcium hydroxide solution for 168 h, 4 mol/L sodium chloride was added and the samples were immersed for another 12 h before conducting the EIS experiment, as shown in Figure 3. Evidently, all steels exhibit similar corrosion behaviors. The Nyquist plot is characterized by a capacitive arc with an extremely large radius, and two time constants exist in the Bode plot. The order of the capacitive arc radius, phase angle peak value, and low frequency impedance modulus is also 10Cr > 5Cr > 0Cr, indicating that the corrosion resistance gradually increases with the Cr content. Comparing Figure 2d with Figure 3d, it can be seen that as the chloride concentration increases, the charge transfer resistance of the steels decreases, that is, the corrosion rate increases. The R_ct_ of 0Cr is 5.9 × 10^3^ Ω·cm^2^. The R_ct_ of 5Cr is 2.24 × 10^4^ Ω·cm^2^, which is 3.80 times higher than that of 0Cr. The R_ct_ of 10Cr is 5.98 × 10^4^ Ω·cm^2^, which is 2.67 times higher than that of 5Cr. Different from the 0.6 mol/L NaCl environment, under the extremely aggressive corrosive condition with 4 mol/L NaCl, the improvement efficiency of the corrosion rate of 5Cr compared with 0Cr is slightly higher than that of 10Cr compared with 5Cr. This indicates that in the conventional marine engineering concrete environment, the improvement in corrosion resistance of 5Cr compared with 0Cr is relatively small, while the improvement in corrosion resistance of 10Cr compared with 5Cr is significant. In a concrete environment with an extremely high chloride concentration, the improvement in corrosion resistance of 5Cr compared with 0Cr is more obvious.

### 3.3. Defect Density of the Passive Film

**Figure 4:**
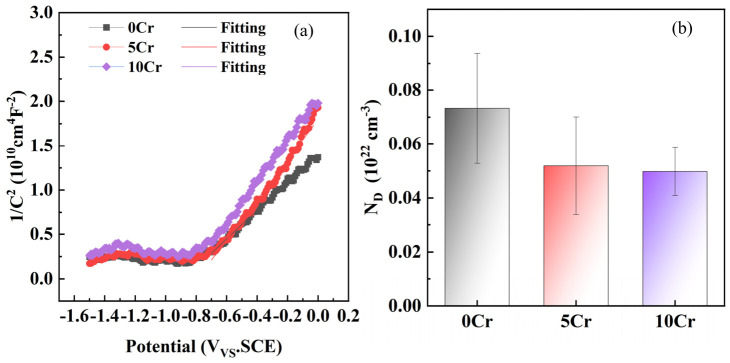
Mott–Schottky curves (**a**) and donor density (**b**) of the three types of steels after immersing in a saturated calcium hydroxide solution for 168 h.

After three steels with different Cr contents were immersed in a saturated calcium hydroxide solution for 168 h, Mott–Schottky experiments were carried out, and the results are shown in Figure 4. Evidently, there is a distinct inflection point at approximately −0.8 V_SCE_. Before the inflection point, the change in 1/C^2^ with potential is relatively gentle, corresponding to the depletion layer characteristics of the space charge region of the passive film. After the inflection point, 1/C^2^ increases linearly with the positive shift in the potential, which conforms to the law of the Mott–Schottky equation for n-type semiconductors [25]. Therefore, the surface passive films of the three types of steels are all n-type semiconductors, with free electrons as the main charge carriers for charge transfer and oxygen vacancies as the dominant defects. Even the 10Cr does not exhibit p-type semiconductor characteristics. With the increase in the Cr content, the curve moves upward as a whole. The 1/C^2^ value of the 10Cr is the highest, followed by 5Cr, and 0Cr is the lowest. This indicates that the space charge layer capacitance of the passive film of high-Cr steel is smaller, and the film density is higher. The Mott–Schottky formula for n-type semiconductors [26] is:

(3) $$\frac{1}{C^{2}}=\frac{1}{C_{H}^{2}}+\frac{2}{\varepsilon \varepsilon_{0}eN_{D}}(V-V_{\mathrm{fb}}-\frac{\mathrm{kT}}{e})$$

From Figure 4b, the N_D_ (donor density) of 0Cr is the highest, while that of 10Cr is the lowest. Therefore, Cr doping can effectively suppress the generation of oxygen vacancy defects within the passive film and reduce the density of free electron charge carriers. Combined with reference [24], the Cr is oxidized to form inert Cr-based products such as Cr_2_O_3_ and Cr(OH)_3_, which fill the oxygen vacancies and grain boundaries of the Fe-based passive film, forming a composite passive structure. Without changing the n-type semiconductor characteristics of the film layer, this improves the density and integrity of the film layer, hinders the diffusion of chloride ions into the film, and simultaneously optimizes the semiconductor stability of the passive film and its resistance to chloride-induced corrosion.

### 3.4. Chemical Composition of the Passive Film

**Figure 5:**
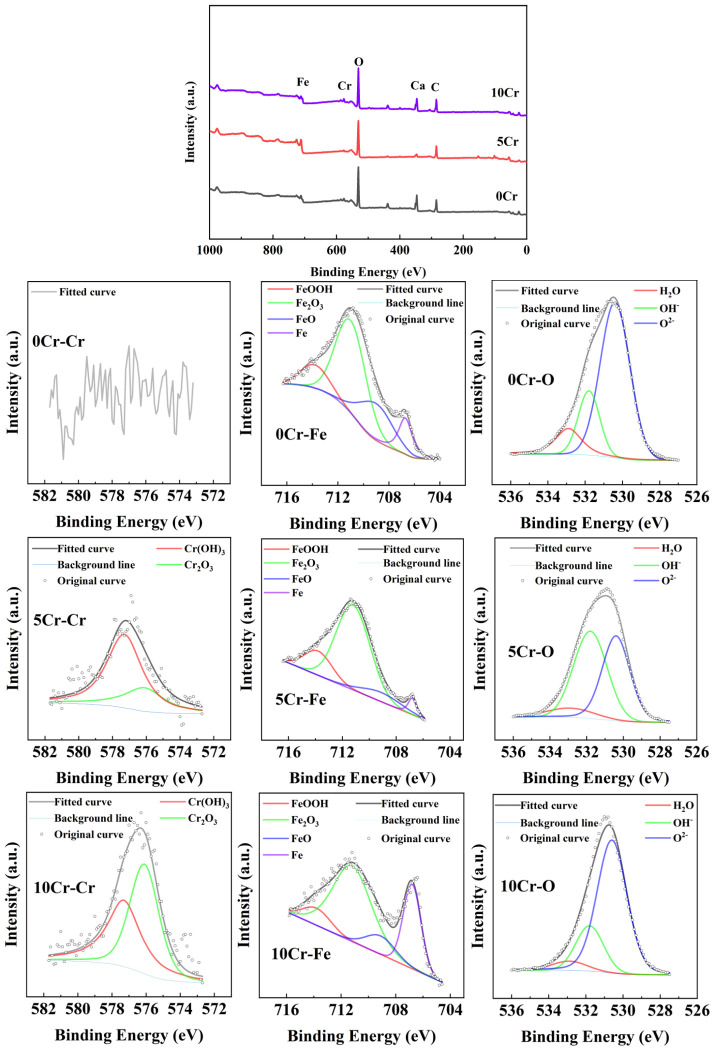

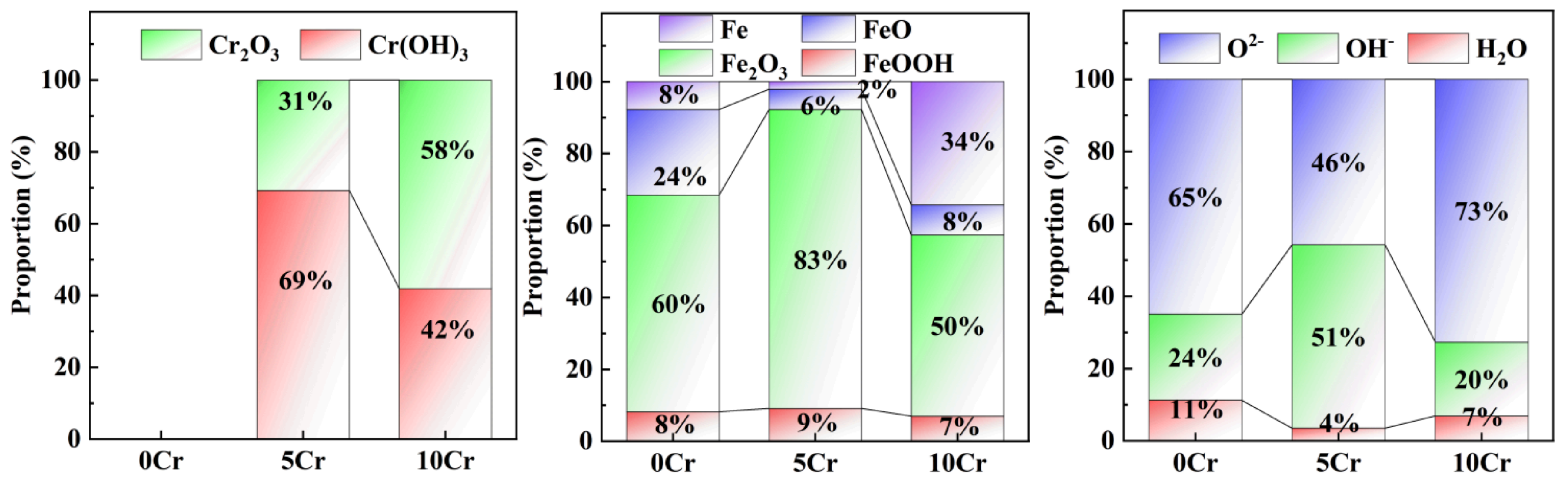
XPS spectra of the three steels after immersion in a saturated calcium hydroxide solution for 168 h.

Figure 5 shows the XPS spectra of the three types of steels immersed in a chloride-free saturated calcium hydroxide solution for 168 h. Evidently, in the high-resolution XPS spectra of the Fe, the Fe^0^ peak can be detected, indicating that the thickness of the passive film is less than the detection thickness of XPS, approximately 5 nm. The area ratios of the Fe^0^ peak for 0Cr, 5Cr, 10Cr are 8%, 6%, and 34%, respectively. Therefore, the passive film of 5Cr is slightly thicker than that of 0Cr, meaning that a small amount of Cr alloying increases the thickness of the passive film. However, the passive film of 10Cr is significantly thinner, about 28.26% less than that of 0Cr. This is related to the formation of a dense and stable passive film on 10Cr, which hinders its further oxidation and thickening. It is generally believed that the passive film of steels in the simulated concrete pore solution consists of two layers. The inner layer is composed of Fe^2+^ substances, and the outer layer is mainly Fe^3+^ substances. The inner layer is the main component that prevents the damage and depassivation of passive film. The phase fraction of various Fe phases shows no distinct regular trend for 0Cr, 5Cr, and 10Cr, which is probably affected by Cr oxide/hydroxide. In the high-resolution XPS spectra of the O element, three chemical states of O^2−^, OH^−^, and H_2_O are detected. The proportions of O^2−^ in 0Cr, 5Cr, and 10Cr are 65%, 46%, and 73%, respectively. This indicates that 10Cr significantly promotes the growth of dense oxides and inhibits the growth of loose hydroxides in the passive film. The passive film of 0Cr is mainly composed of Fe and O elements. In addition to Fe and O elements, a large amount of Cr is also present in the passive films of 5Cr and 10Cr. Since the sensitivity factors of the Cr and the Fe in XPS are relatively close, the area ratio of Fe to Cr is used to roughly evaluate the Cr/Fe ratio. The Cr/Fe ratios of 5Cr and 10Cr are 0.47 and 0.46 respectively, indicating that the Cr is enriched in the passive film and the concentration difference is not significant. The Cr in the Cr-alloyed steel mainly exhibits two peaks: Cr(OH)_3_ and Cr_2_O_3_. Compared with 5Cr, the peak intensity of Cr(OH)_3_ in 10Cr decreases, while the peak intensity of Cr_2_O_3_ increases. It is generally believed that Cr_2_O_3_ has low electrical conductivity and is the key to the self-repair and stability maintenance of the passive film. Therefore, the passive film of 10Cr has higher stability due to the significant increase in the content of Cr_2_O_3_.

**Figure 6:**
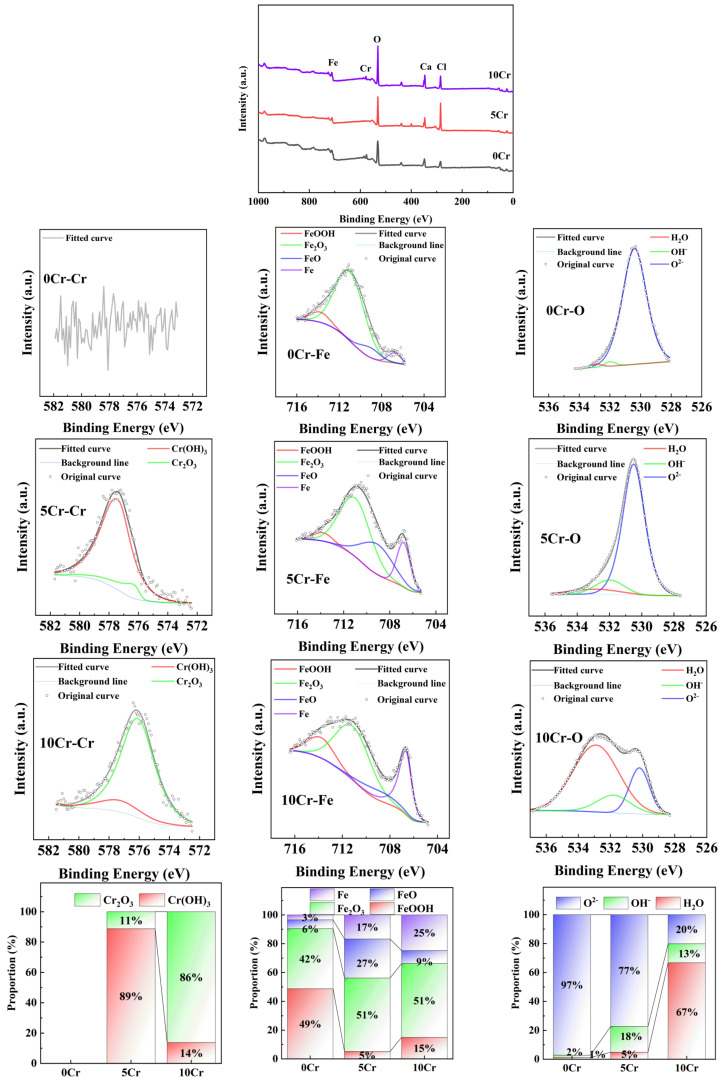
XPS spectra of the three steels after immersing in a saturated calcium hydroxide solution for 168 h and then further immersing in a solution containing 0.6 mol/L NaCl for 24 h.

Figure 6 shows the XPS spectra of the three types of steels after being immersed in a saturated calcium hydroxide solution for 7 days, followed by the addition of 0.6 mol/L NaCl and further immersion for 24 h. The area ratios of the Fe^0^ peak for 0Cr, 5Cr, 10Cr are 3%, 17%, and 25% respectively. Therefore, the passive film of 10Cr is still the thinnest, about 22.68% less than that of 0Cr. Compared with the passive films formed in a chloride-free environment, the passive films of 0Cr and 10Cr thicken under the action of chloride ions, while the passive film of 5Cr thins in the chloride-ion environment. The Fe^2+^/Fe^3+^ ratios of 0Cr, 5Cr, and 10Cr are 0.06, 0.37, and 0.12 respectively, indicating that Cr significantly increases the proportion of the dense Fe^2+^ layer in the passive film and inhibits the oxidation of the Fe phase in the passive film. Compared with the chloride-free environment, the Fe^2+^/Fe^3+^ ratio of 0Cr decreases significantly, while that of 5Cr increases significantly, and the ratio of 10Cr remains basically unchanged. Considering the changes in Fe^0^ and Fe^2+^/Fe^3+^, it can be suggested that in the chloride-contaminated concrete environment, the inner Fe^2+^ layer of the passive film on 0Cr has insufficient protection and is continuously oxidized, causing the loose outer layer rich in Fe^3+^ to thicken significantly, resulting in the thickening of the entire passive film. In contrast, the inner Fe^2+^ layer of the passive film on 5Cr has enhanced protection and remains stable under chloride attack, while the loose outer layer rich in Fe^3+^ thins significantly, resulting in the thinning of the entire passive film. Moreover, the Fe oxide/hydroxide phase of 10Cr is less affected by chloride ions. The proportions of Cr_2_O_3_ in 5Cr and 10Cr are 11% and 86% respectively. Evidently, under the action of chloride ions, the Cr phase in 5Cr transforms into hydroxide, resulting in a decrease in protection. However, the proportion of Cr_2_O_3_ in 10Cr increases, further enhancing the protection of the passive film.

## 4. Discussion

In the alkaline concrete environment, the passive film formed on the surface of steel reinforcements has a double-layer structure. The inner layer is mainly composed of incompletely oxidized mixed oxides Fe^2+^, and the outer layer is mainly highly oxidized Fe^3+^. This crystal structure is loose, the lattice arrangement is disordered, with obvious lattice distortion, and the overall density of the film layer is low, making it difficult to form an effective corrosion protection barrier. With the Cr alloying of steels, Cr preferentially participates in the passive film, forming a Cr-rich double-layer passive film on the steel surface. The inner layer is Cr oxide/hydroxides, and the outer layer is Fe oxide/hydroxides. The degree of order of crystal arrangement is greatly improved, the degree of lattice distortion is significantly reduced, and the spatial-structure stability of the film layer is obviously enhanced.

In a chloride-contaminated concrete environment, chloride ions will adsorb on the surface of the steel, causing local acidification. This weakens the Fe-O bonds in the passive film, promoting the dissociation of Fe^3+^ in the outer layer of the passive film. The resulting cation vacancies are filled by H^+^, forming -OH groups, which leads to the hydroxylation of the surrounding Fe_2_O_3_. As a result, the ratio of FeOOH/Fe_2_O_3_ in the passive film increases. This accelerates the inward transport of chloride ions and significantly reduces the corrosion resistance of the steel [27,28]. In contrast, the Cr-O bond is relatively stable, so Cr_2_O_3_ can exist stably in a chloride environment. Compared with 5Cr, 10Cr preferentially forms more Cr_2_O_3_. As the main barrier of the passive film, Cr_2_O_3_ can reduce the generation of cation vacancies, prevent the formation of more unstable hydroxides, and thus increase the stability of the passive film. Cr_2_O_3_ possesses excellent self-repairing capability. When the chloride concentration is extremely high and exceeds the threshold value, the self-repairing ability fails, thereby inducing corrosion [29,30]. As observed in this study, when the mass fraction of Cr in the steel is 5.2 wt.%, the Cr(OH)_3_ crystal is the dominant Cr phase in the passive film. It is difficult to form a dense and regular crystal structure, and the protective performance of its passive film is poor. At this time, there are a large number of oxygen vacancy defects in the passive film. These defects act as fast channels for the penetration of corrosive media, accelerating the electron transfer inside the film layer, making the integrity and density of the film layer easy to be damaged, and the protection ability weak. However, when the mass fraction of Cr reaches 10.23 wt.%, the proportion of Cr_2_O_3_ increases significantly. Especially in the chloride environment, the Cr_2_O_3_ content is as high as over 80%. This Cr_2_O_3_ covering layer plays a role in regulating corrosion resistance, effectively improving the chemical stability of the passive film, greatly reducing the density of oxygen vacancy defects, and reducing the active sites where the film layer is dissolved and broken through by corrosive media. In terms of corrosion resistance, the critical chloride concentrations of 0Cr, 5Cr, and 10Cr are 0.63 mol/L, 0.81 mol/L, and 1.56 mol/L respectively. In a 0.6 mol/L chloride-containing concrete environment, the charge transfer resistances of 0Cr, 5Cr, and 10Cr are 4.14 × 10^5^ Ω·cm^2^, 5.77 × 10^5^ Ω·cm^2^, and 6.34 × 10^6^ Ω·cm^2^ respectively. The above results all indicate that as a low-alloy steel, the corrosion resistance enhancement effect of 5Cr is far lower than that of 10Cr in the conventional marine chloride-contaminated concrete.

## 5. Conclusions

In this study, electrochemical tests and physicochemical characterization techniques were employed to explore the chloride-induced corrosion behaviors and the passive film properties of low-Cr and high-Cr steel bars in simulated concrete pore solutions. The results indicate that the characteristics of Cr in regulating the microstructure and corrosion resistance of the passive film are significantly related to the Cr content and chloride concentration. In a common marine chloride-contaminated environment, the corrosion resistance enhancement effect of 5Cr is not remarkable, while that of 10Cr is significantly improved. In an environment with an extremely high chloride concentration, the corrosion resistance enhancement effect of 5Cr is obvious.

In a saturated calcium hydroxide solution with the gradual addition of chloride, the critical chloride concentrations of 0Cr, 5Cr, and 10Cr are 0.63 mol/L, 0.81 mol/L, and 1.56 mol/L, respectively. The improvement in corrosion resistance of 10Cr relative to 5Cr is the most significant. In an alkaline environment containing 0.6 mol/L NaCl, the charge transfer resistances of 0Cr, 5Cr, and 10Cr are 4.14 × 10^5^ Ω·cm^2^, 5.77 × 10^5^ Ω·cm^2^, and 6.34 × 10^6^ Ω·cm^2^, respectively. The improvement in corrosion resistance of 10Cr relative to 5Cr is the most significant.

Mott–Schottky analysis shows that in an alkaline environment, the passive films on the surfaces of carbon steel and Cr-alloyed steels all exhibit n-type semiconductor characteristics, with free electrons as the main charge carriers and oxygen vacancies as the main point defects. Cr markedly reduces the donor charge carrier concentration, thereby improving the compactness and protectiveness of the passive film.

XPS analysis indicates that Cr(OH)_3_ is the main substance in the passive film of low-Cr steel, while Cr_2_O_3_ is the main substance in the passive film of high-Cr steels. Cr(OH)_3_ is unable to provide good barrier effects within a chloride environment, whereas Cr_2_O_3_ effectively hinders the penetration of chloride ions and the oxidation of Fe^2+^, thereby enhancing the protective efficiency of the passive film.

In conclusion, 10.23 wt.% of Cr-alloyed steel exhibits distinctly better corrosion resistance than that with a lower weight percentage within a conventional chloride-contaminated concrete environment. Accordingly, the application of high-Cr steel in marine engineering possesses important engineering value for improving the chloride corrosion resistance of steel reinforcements and prolonging the service life of concrete structures.

However, the present study still has certain limitations. First, there is an obvious difference in carbon content between carbon steel and Cr-alloyed steels. The carbon content of carbon steel can be as high as 0.25 wt.% to meet the requirement of mechanical strength. A large amount of cementite precipitated in the steel matrix tends to form corrosion micro-galvanic couples, which adversely interfere with the corrosion resistance of the substance and, to a certain extent, mask the intrinsic law of the individual Cr element on corrosion resistance. Second, the critical chloride concentration obtained in this work is measured in a simulated concrete pore solution. In fact, testing the chloride threshold in real concrete is of greater practical application significance. Therefore, further research will be carried out as a follow-up to provide more accurate theoretical basis and data support for the material selection of corrosion-resistant steel rebars and anticorrosion design in marine engineering.

## Figures and Tables

**Table 1:** Chemical composition of Cr-alloyed steels.

Steel Reinforcement	C (wt.%)	Si (wt.%)	Mn (wt.%)	P (wt.%)	S (wt.%)	Cr (wt.%)
0Cr	0.25	0.58	1.53	0.004	0.003	0.012
5Cr	0.091	0.26	1.37	0.02	0.011	5.19
10Cr	0.003	0.46	1.48	0.004	0.004	10.23

**Table 2:** Fitted values of the EIS in Figure 2 and Figure 3.

		Q_f_ Ω^−1^S^n^cm^−2^	n_f_	R_f_ Ω·cm^2^	Q_dl_ Ω^−^1 ·Sn·cm^−2^	n_dl_	R_ct_ Ω·cm^2^	C_f_ μF·cm^−2^	C_dl_ μF·cm^−2^
Ca(OH)_2_ + 0.6 mol/L NaCl	0Cr	4.17 × 10^−5^ ±1.51 × 10^−6^	0.921 ±0.002	9.70 × 10^3^ ±1.35 × 10^2^	1.22 × 10^−5^ ±6.07 × 10^−6^	0.595 ±0.049	4.14 × 10^5^ ±1.71 × 10^4^	3.86 × 10^−5^	5.70 × 10^−5^
	5Cr	2.86 × 10^−5^ ±1.79 × 10^−5^	0.960 ±0.056	1.52 × 10^5^ ±2.56 × 10^4^	1.68 × 10^−5^ ±1.36 × 10^−5^	0.673 ±0.219	5.77 × 10^5^ ±5.38 × 10^4^	3.04 × 10^−5^	5.31 × 10^−5^
	10Cr	1.44 × 10^−5^ ±2.85 × 10^−6^	0.916 ±0.020	2.83 × 10^5^ ±7.11 × 10^4^	1.64 × 10^−5^ ±8.78 × 10^−6^	0.930 ±0.097	6.34 × 10^6^ ±6.48 × 10^5^	1.72 × 10^−5^	8.27 × 10^−6^
Ca(OH)_2_ + 4 mol/L NaCl	0Cr	7.43 × 10^−5^ ±1.22 × 10^−5^	0.899 ±0.022	2.28 × 10^3^ ±1.07 × 10^3^	4.39 × 10^−4^ ±1.06 × 10^−4^	0.636 ±0.057	5.90 × 10^3^ ±7.46 × 10^2^	6.09 × 10^−5^	7.96 × 10^−6^
	5Cr	2.84 × 10^−5^ ±4.73 × 10^−5^	0.833 ±0.211	8.64 × 10^6^ ±1.50 × 10^6^	1.02 × 10^−4^ ±6.15 × 10^−4^	0.504 ±0.457	2.24 × 10^4^ ±3.28 × 10^4^	8.60 × 10^−5^	7.48 × 10^−6^
	10Cr	6.78 × 10^−5^ ±5.87 × 10^−5^	0.787 ±0.185	2.74 ±5.33 × 10^−1^	4.62 × 10^−5^ ±3.15 × 10^−5^	0.954 ±0.072	5.98 × 10^4^ ±3.05 × 10^4^	6.63 × 10^−6^	2.01 × 10^−5^

## Data Availability

The original contributions presented in this study are included in the article. Further inquiries can be directed to the corresponding author.
